# 5-Chloro-2-(4-meth­oxy­phen­yl)-1,3-benzo­thia­zole

**DOI:** 10.1107/S1600536813001955

**Published:** 2013-02-09

**Authors:** Sammer Yousuf, Shazia Shah, Nida Ambreen, Khalid M. Khan, Shakil Ahmad

**Affiliations:** a H.E.J. Research Institute of Chemistry, International Center for Chemical and Biological Sciences, University of Karachi, Karachi 75270, Pakistan

## Abstract

In the title compound, C_14_H_10_ClNOS, the dihedral angle between the benzothia­zole ring system and the meth­oxy-substituted benzene ring is 8.76 (16)°. In the crystal, mol­ecules are stacked in columns along the *c* axis and no significant inter­molecular inter­actions are observed.

## Related literature

For the biological activity of benzothia­zole compounds, see: Chohan *et al.* (2003 ▶); Khan *et al.* (2011 ▶); Hutchinson *et al.* (2002 ▶); Burger & Sawhney (1968 ▶); Palmer *et al.* (1971 ▶). For related structures, see: Yousuf *et al.* (2012*a* ▶,*b* ▶).
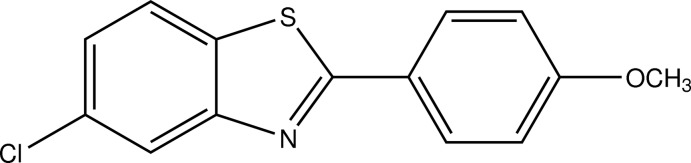


## Experimental

### 

#### Crystal data

C_14_H_10_ClNOS
*M* *_r_* = 275.74
Orthorhombic, 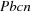

*a* = 29.0274 (16) Å
*b* = 14.5512 (8) Å
*c* = 5.8686 (3) Å
*V* = 2478.8 (2) Å^3^
*Z* = 8
Mo *K*α radiation
μ = 0.46 mm^−1^
*T* = 273 K
0.37 × 0.22 × 0.10 mm

#### Data collection

Bruker SMART APEX CCD area-detector diffractometer
Absorption correction: multi-scan (*SADABS*; Bruker, 2000 ▶) *T*_min_ = 0.848, *T*_max_ = 0.955
13397 measured reflections
2299 independent reflections
1965 reflections with *I* > 2σ(*I*)
*R*_int_ = 0.052

#### Refinement

*R*[*F* ^2^ > 2σ(*F* ^2^)] = 0.076
*wR*(*F* ^2^) = 0.208
*S* = 1.15
2299 reflections
164 parameters
H-atom parameters constrained
Δρ_max_ = 0.63 e Å^−3^
Δρ_min_ = −0.37 e Å^−3^

### 

Data collection: *SMART* (Bruker, 2000 ▶); cell refinement: *SAINT* (Bruker, 2000 ▶); data reduction: *SAINT*; program(s) used to solve structure: *SHELXS97* (Sheldrick, 2008 ▶); program(s) used to refine structure: *SHELXL97* (Sheldrick, 2008 ▶); molecular graphics: *SHELXTL* (Sheldrick, 2008 ▶); software used to prepare material for publication: *SHELXTL*, *PARST* (Nardelli, 1996 ▶) and *PLATON* (Spek, 2009 ▶).

## Supplementary Material

Crystal structure: contains datablock(s) global, I. DOI: 10.1107/S1600536813001955/is5237sup1.cif

Structure factors: contains datablock(s) I. DOI: 10.1107/S1600536813001955/is5237Isup2.hkl

Supplementary material file. DOI: 10.1107/S1600536813001955/is5237Isup3.cml

Additional supplementary materials:  crystallographic information; 3D view; checkCIF report

## Figures and Tables

**Fig. 1.**
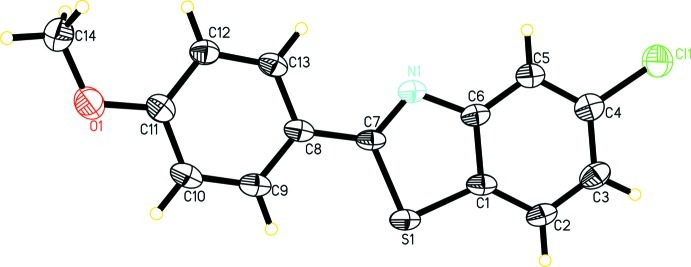
The molecular structure of title compound with displacement ellipsoids drawn at 30% probability level.

**Fig. 2.**
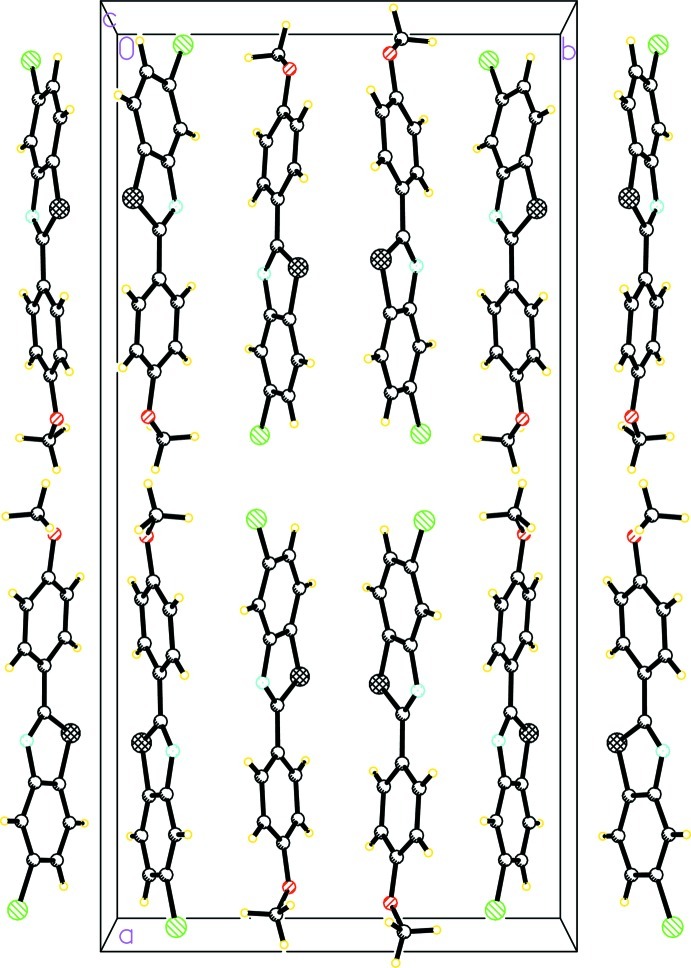
A crystal packing diagram of the title compound, viewed along the c axis.

## supplementary crystallographic information

### Comment

Benzothiazole is a well known class of organic compounds with a diverse range of biological activities (Khan *et al.*, 2011; Chohan *et al.*, 2003, Hutchinson *et al.*, 2002; Burger & Sawhney, 1968; Palmer *et al.*, 1971). The title compound is a methoxy phenyl derivative of benzothiazole synthesized as a part of our ongoing project to synthesize bioactive hetereocyclic compounds.

The crystal structure of title compound (Fig. 1), C_16_H_14_ClNOS, is similar to that our previously published 5-chloro-2-(3,4,5-trimethoxyphenyl)-1,3-benzothiazole (Yousuf *et al.*, 2012*b*) with the difference that the 3,4,5-trimethoxyphenyl ring is replaced by the 4-methoxyphenyl phenyl ring. The dihedral angle between planner benzothiazole (S1/N1/C1–C7) and methoxy phenyl rings (C8–C13) is 8.76 (16)°. The bond lengths and angle are similar as in previously published benzothiazole compounds (Yousuf *et al.*, 2012*a*,*b*). In the crystal structure the molecules having plane of mirror are arranged in a two-diminesional manner along *a* and *c* axes (Fig. 2).

### Experimental

A mixture of 2-amino-4-cholorobenzenethiol (0.159 g, 1 mmol), 4-methoxybenzaldehyde (0.136 g, 1 mmol), sodium metabisulfite (0.2 g) and *N*,*N*-dimethylformamide (10 ml) was refluxed for 2 hrs in a round-bottomed flask. The completion of reaction was monitored by TLC and cool to room temperature followed by addition of cold water to obtain white precipitates. Crystallization from ethanol afforded pure crystal of 5-chloro-2-(4-methoxyphenyl) benzothiazole (yield 0.223 g, 81.1%) found suitable for X-ray diffraction studies.

### Refinement

H atoms of phenyl and methyl groups were positioned geometrically with C—H = 0.93 and 0.96 Å, respectively, and constrained to ride on their parent atoms with *U*_iso_(H) = 1.2*U*_eq_(C_phenyl_) and 1.5*U*_eq_(C_methyl_). A rotating group model was applied to the methyl group.

### Crystal data

**Table:**

C_14_H_10_ClNOS	*F*(000) = 1136
*M**_r_* = 275.74	*D*_x_ = 1.478 Mg m^−^^3^
Orthorhombic, *P**b**c**n*	Mo *K*α radiation, λ = 0.71073 Å
Hall symbol: -P 2n 2ab	Cell parameters from 3307 reflections
*a* = 29.0274 (16) Å	θ = 2.5–26.3°
*b* = 14.5512 (8) Å	µ = 0.46 mm^−^^1^
*c* = 5.8686 (3) Å	*T* = 273 K
*V* = 2478.8 (2) Å^3^	Block, colorles
*Z* = 8	0.37 × 0.22 × 0.10 mm

### Data collection

**Table:**

Bruker SMART APEX CCD area-detector diffractometer	2299 independent reflections
Radiation source: fine-focus sealed tube	1965 reflections with *I* > 2σ(*I*)
Graphite monochromator	*R*_int_ = 0.052
ω scan	θ_max_ = 25.5°, θ_min_ = 1.6°
Absorption correction: multi-scan (*SADABS*; Bruker, 2000)	*h* = −34→34
*T*_min_ = 0.848, *T*_max_ = 0.955	*k* = −17→17
13397 measured reflections	*l* = −6→7

### Refinement

**Table:**

Refinement on *F*^2^	Primary atom site location: structure-invariant direct methods
Least-squares matrix: full	Secondary atom site location: difference Fourier map
*R*[*F*^2^ > 2σ(*F*^2^)] = 0.076	Hydrogen site location: inferred from neighbouring sites
*wR*(*F*^2^) = 0.208	H-atom parameters constrained
*S* = 1.15	*w* = 1/[σ^2^(*F*_o_^2^) + (0.0983*P*)^2^ + 3.3682*P*] where *P* = (*F*_o_^2^ + 2*F*_c_^2^)/3
2299 reflections	(Δ/σ)_max_ < 0.001
164 parameters	Δρ_max_ = 0.63 e Å^−^^3^
0 restraints	Δρ_min_ = −0.37 e Å^−^^3^

### Special details

**Table:**

Geometry. All e.s.d.'s (except the e.s.d. in the dihedral angle between two l.s. planes) are estimated using the full covariance matrix. The cell e.s.d.'s are taken into account individually in the estimation of e.s.d.'s in distances, angles and torsion angles; correlations between e.s.d.'s in cell parameters are only used when they are defined by crystal symmetry. An approximate (isotropic) treatment of cell e.s.d.'s is used for estimating e.s.d.'s involving l.s. planes.
Refinement. Refinement of *F*^2^ against ALL reflections. The weighted *R*-factor *wR* and goodness of fit *S* are based on *F*^2^, conventional *R*-factors *R* are based on *F*, with *F* set to zero for negative *F*^2^. The threshold expression of *F*^2^ > σ(*F*^2^) is used only for calculating *R*-factors(gt) *etc*. and is not relevant to the choice of reflections for refinement. *R*-factors based on *F*^2^ are statistically about twice as large as those based on *F*, and *R*- factors based on ALL data will be even larger.

### Fractional atomic coordinates and isotropic or equivalent isotropic displacement parameters (Å^2^)

**Table:**

	*x*	*y*	*z*	*U*_iso_*/*U*_eq_	
Cl1	0.04504 (4)	0.17240 (11)	−0.0360 (2)	0.0787 (5)	
S1	0.21904 (4)	0.08514 (7)	0.52664 (16)	0.0494 (4)	
O1	0.43838 (11)	0.1057 (2)	0.1916 (6)	0.0656 (9)	
N1	0.21907 (12)	0.1613 (2)	0.1301 (5)	0.0437 (8)	
C1	0.16642 (15)	0.1048 (2)	0.4009 (6)	0.0450 (9)	
C2	0.12234 (17)	0.0857 (3)	0.4807 (7)	0.0502 (10)	
H2A	0.1179	0.0592	0.6231	0.060*	
C3	0.08560 (16)	0.1072 (3)	0.3433 (7)	0.0556 (11)	
H3A	0.0558	0.0946	0.3927	0.067*	
C4	0.09246 (15)	0.1475 (3)	0.1304 (8)	0.0530 (10)	
C5	0.13595 (15)	0.1688 (3)	0.0488 (7)	0.0471 (9)	
H5A	0.1400	0.1968	−0.0922	0.057*	
C6	0.17346 (14)	0.1466 (2)	0.1872 (6)	0.0420 (9)	
C7	0.24620 (14)	0.1318 (2)	0.2888 (6)	0.0409 (9)	
C8	0.29667 (14)	0.1305 (2)	0.2692 (6)	0.0420 (9)	
C9	0.32493 (16)	0.0941 (3)	0.4380 (7)	0.0511 (10)	
H9A	0.3118	0.0730	0.5727	0.061*	
C10	0.37175 (16)	0.0885 (3)	0.4104 (7)	0.0535 (11)	
H10A	0.3901	0.0646	0.5261	0.064*	
C11	0.39169 (15)	0.1187 (3)	0.2077 (7)	0.0487 (10)	
C12	0.36468 (15)	0.1576 (3)	0.0376 (7)	0.0479 (9)	
H12A	0.3780	0.1795	−0.0959	0.057*	
C13	0.31762 (15)	0.1631 (2)	0.0709 (7)	0.0461 (9)	
H13A	0.2994	0.1893	−0.0421	0.055*	
C14	0.46063 (17)	0.1316 (4)	−0.0114 (9)	0.0717 (14)	
H14A	0.4926	0.1149	−0.0032	0.108*	
H14B	0.4580	0.1969	−0.0316	0.108*	
H14C	0.4465	0.1007	−0.1379	0.108*	

### Atomic displacement parameters (Å^2^)

**Table:**

	*U*^11^	*U*^22^	*U*^33^	*U*^12^	*U*^13^	*U*^23^
Cl1	0.0538 (7)	0.1036 (11)	0.0787 (9)	0.0116 (7)	0.0019 (6)	0.0106 (7)
S1	0.0692 (7)	0.0454 (6)	0.0336 (5)	−0.0036 (5)	0.0005 (4)	0.0084 (4)
O1	0.0598 (19)	0.072 (2)	0.065 (2)	0.0054 (15)	−0.0097 (16)	0.0031 (16)
N1	0.058 (2)	0.0384 (16)	0.0347 (16)	−0.0028 (14)	0.0022 (15)	0.0019 (13)
C1	0.069 (3)	0.0347 (18)	0.0308 (18)	−0.0014 (17)	0.0019 (18)	0.0005 (14)
C2	0.068 (3)	0.044 (2)	0.039 (2)	−0.0038 (19)	0.0129 (19)	0.0029 (16)
C3	0.066 (3)	0.051 (2)	0.050 (2)	−0.004 (2)	0.020 (2)	−0.0059 (19)
C4	0.061 (3)	0.046 (2)	0.052 (2)	0.0044 (18)	0.007 (2)	−0.0042 (18)
C5	0.061 (2)	0.0386 (19)	0.042 (2)	0.0006 (17)	0.0043 (19)	−0.0007 (16)
C6	0.061 (2)	0.0313 (16)	0.0334 (18)	−0.0018 (16)	0.0071 (17)	−0.0023 (14)
C7	0.064 (2)	0.0315 (17)	0.0269 (17)	−0.0024 (16)	0.0010 (17)	−0.0002 (14)
C8	0.065 (2)	0.0298 (16)	0.0314 (18)	−0.0007 (15)	−0.0052 (17)	−0.0006 (14)
C9	0.072 (3)	0.045 (2)	0.036 (2)	−0.0032 (19)	−0.0038 (19)	0.0056 (16)
C10	0.071 (3)	0.047 (2)	0.043 (2)	0.0017 (19)	−0.016 (2)	0.0052 (17)
C11	0.056 (2)	0.043 (2)	0.047 (2)	0.0021 (17)	−0.0078 (19)	−0.0040 (17)
C12	0.059 (2)	0.042 (2)	0.043 (2)	−0.0012 (18)	−0.0009 (18)	0.0047 (16)
C13	0.061 (2)	0.0376 (19)	0.040 (2)	0.0003 (17)	−0.0088 (18)	0.0040 (15)
C14	0.055 (3)	0.092 (4)	0.068 (3)	−0.007 (3)	0.001 (2)	0.004 (3)

### Geometric parameters (Å, º)

**Table:**

Cl1—C4	1.726 (5)	C5—H5A	0.9300
S1—C1	1.720 (4)	C7—C8	1.470 (6)
S1—C7	1.741 (4)	C8—C9	1.391 (5)
O1—C11	1.372 (5)	C8—C13	1.396 (5)
O1—C14	1.407 (5)	C9—C10	1.371 (7)
N1—C7	1.293 (5)	C9—H9A	0.9300
N1—C6	1.382 (5)	C10—C11	1.394 (6)
C1—C2	1.391 (6)	C10—H10A	0.9300
C1—C6	1.409 (5)	C11—C12	1.390 (5)
C2—C3	1.373 (7)	C12—C13	1.382 (6)
C2—H2A	0.9300	C12—H12A	0.9300
C3—C4	1.394 (6)	C13—H13A	0.9300
C3—H3A	0.9300	C14—H14A	0.9600
C4—C5	1.386 (6)	C14—H14B	0.9600
C5—C6	1.396 (6)	C14—H14C	0.9600
			
C1—S1—C7	89.62 (18)	C9—C8—C13	117.8 (4)
C11—O1—C14	118.4 (4)	C9—C8—C7	122.5 (4)
C7—N1—C6	110.9 (3)	C13—C8—C7	119.7 (3)
C2—C1—C6	121.3 (4)	C10—C9—C8	121.5 (4)
C2—C1—S1	129.8 (3)	C10—C9—H9A	119.2
C6—C1—S1	109.0 (3)	C8—C9—H9A	119.2
C3—C2—C1	118.2 (4)	C9—C10—C11	119.6 (4)
C3—C2—H2A	120.9	C9—C10—H10A	120.2
C1—C2—H2A	120.9	C11—C10—H10A	120.2
C2—C3—C4	120.7 (4)	O1—C11—C12	124.4 (4)
C2—C3—H3A	119.6	O1—C11—C10	115.2 (4)
C4—C3—H3A	119.6	C12—C11—C10	120.5 (4)
C5—C4—C3	122.3 (4)	C13—C12—C11	118.7 (4)
C5—C4—Cl1	118.9 (3)	C13—C12—H12A	120.7
C3—C4—Cl1	118.8 (3)	C11—C12—H12A	120.7
C4—C5—C6	117.2 (4)	C12—C13—C8	121.9 (4)
C4—C5—H5A	121.4	C12—C13—H13A	119.0
C6—C5—H5A	121.4	C8—C13—H13A	119.0
N1—C6—C5	124.7 (3)	O1—C14—H14A	109.5
N1—C6—C1	114.9 (4)	O1—C14—H14B	109.5
C5—C6—C1	120.3 (4)	H14A—C14—H14B	109.5
N1—C7—C8	123.7 (3)	O1—C14—H14C	109.5
N1—C7—S1	115.5 (3)	H14A—C14—H14C	109.5
C8—C7—S1	120.6 (3)	H14B—C14—H14C	109.5
			
C7—S1—C1—C2	179.4 (4)	C1—S1—C7—N1	1.1 (3)
C7—S1—C1—C6	−0.7 (3)	C1—S1—C7—C8	−174.8 (3)
C6—C1—C2—C3	1.2 (6)	N1—C7—C8—C9	−176.7 (3)
S1—C1—C2—C3	−178.9 (3)	S1—C7—C8—C9	−1.1 (5)
C1—C2—C3—C4	−0.4 (6)	N1—C7—C8—C13	0.5 (5)
C2—C3—C4—C5	−0.9 (6)	S1—C7—C8—C13	176.0 (3)
C2—C3—C4—Cl1	179.6 (3)	C13—C8—C9—C10	−1.2 (6)
C3—C4—C5—C6	1.3 (6)	C7—C8—C9—C10	176.0 (4)
Cl1—C4—C5—C6	−179.2 (3)	C8—C9—C10—C11	−0.9 (6)
C7—N1—C6—C5	−178.4 (3)	C14—O1—C11—C12	−1.3 (6)
C7—N1—C6—C1	0.7 (4)	C14—O1—C11—C10	177.8 (4)
C4—C5—C6—N1	178.6 (3)	C9—C10—C11—O1	−176.6 (4)
C4—C5—C6—C1	−0.4 (5)	C9—C10—C11—C12	2.5 (6)
C2—C1—C6—N1	−179.9 (3)	O1—C11—C12—C13	177.1 (4)
S1—C1—C6—N1	0.2 (4)	C10—C11—C12—C13	−1.9 (6)
C2—C1—C6—C5	−0.8 (5)	C11—C12—C13—C8	−0.2 (6)
S1—C1—C6—C5	179.3 (3)	C9—C8—C13—C12	1.8 (5)
C6—N1—C7—C8	174.5 (3)	C7—C8—C13—C12	−175.5 (3)
C6—N1—C7—S1	−1.2 (4)		
